# Mid- and long-term results of open repair for chronic type B aortic dissection in endovascular era

**DOI:** 10.1007/s00380-024-02399-1

**Published:** 2024-03-27

**Authors:** Akitoshi Takazawa, Toshihisa Asakura, Osamu Kinoshita, Hiroyuki Nakajima, Akihiro Yoshitake

**Affiliations:** Department of Cardiovascular Surgery, Saitama Medical University International Medical Center, 1397-1 Yamane, Hidaka City, Saitama Japan

**Keywords:** Chronic type B aortic dissection, Open surgical repair, Thoracic endovascular aortic repair, Mid- and long-term outcomes, Crossed-over outcome

## Abstract

Medical management is the standard treatment of chronic type B aortic dissection (CTBAD). However, the roles of open surgical repair (OSR) and thoracic endovascular repair (TEVAR) in patients with CTBAD remain controversial. Thus, this study aimed to assess and compare the mid- and long-term clinical outcomes of OSR via left thoracotomy with that of TEVAR for CTBAD. The data of 85 consecutive patients who underwent surgery for CTBAD from April 2007 to May 2021 were retrospectively reviewed. The patients were divided into two groups: Group G, which included patients who underwent OSR, and Group E, which included patients who underwent TEVAR. Groups G and E comprised 33 and 52 patients, respectively. Preoperative and postoperative computed tomography (CT) studies were retrospectively analyzed for the maximum diameter. The mean duration of the follow-up period was 5.8 years. Operative mortality did not occur. There was no difference in complications, such as stroke (G: 2 vs. E: 0, *p* = 0.30), paraplegia (G: 1 vs. E: 1, *p* = 0.66), and respiratory failure (G: 2, vs. E: 0, *p* = 0.30). The difference in preoperative factors was observed, including the intervals between onset and operation (G; 4.9 years vs. E; 1.9 years, *p* < 0.01), maximum diameter in preoperative CT (G; 59.0 mm vs. E; 50.5 mm, *p* < 0.001), and maximum false lumen diameter (G; 35.5 mm vs. E; 29.0 mm, *p* < 0.01). There was no significant difference in the mid- and long-term survival rates (*p* = 0.49), aorta-related deaths (*p* = 0.33), and thoracic re-intervention rates (*p* = 0.34). Postoperative adverse events occurred in Group E: four cases of retrospective type A aortic dissection, two cases of aorto-bronchial fistula, and one case of aorto-esophagus fistula. Aorta-related death and re-intervention rates crossed over in both groups after seven years postoperatively. Although endovascular repair of CTBAD is less invasive, the rate of freedom from re-intervention was unsatisfactory. Some fatal complications were observed in the endovascular group, and the mid- and long-term outcomes were reversed compared with those in the OSR group. Although OSR is an invasive procedure, it could be performed safely without perioperative complications. OSR has more feasible mid- and long-term outcomes.

## Introduction

Medical management remains the standard treatment modality of type B aortic dissection (TBAD). Different strategies are required for chronic complicated and uncomplicated cases. Although appropriate medical therapy has been administered, dilation of the aneurysm in the subacute or chronic phase has been reported, with a growth rate of 4 mm/year in the thoracic aorta [1]. Particularly, residual dissection particularly persists in patients with acute aortic dissection. In the chronic phase, conventional open surgery or thoracic endovascular aortic repair (TEVAR) should be performed in those with malperfusion, intractable pain, or other severe complications [2–4]. Treatment aim is to prevent aortic-related death in mid- and late-term outcomes. Although open surgery or the endovascular approach has been discussed, their roles in patients with chronic TBAD remain controversial. Thus, this single-center study aimed to assess the mid- and long-term clinical outcomes of conventional open surgery compared with TEVAR for chronic TBAD (CTBAD).

## Materials and methods

### Patients and study design

Data were obtained from the database of Saitama Medical University International Medical Center. The study protocol was approved by our Institutional Ethics Committee (ID: 19–164, September 4, 2019).

The records of 107 consecutive patients who underwent surgery for chronic TBAD at our center between July 2007 and May 2021 were retrospectively reviewed. A total of 22 were excluded because they underwent total aortic arch replacement using the frozen elephant trunk technique. Meanwhile, 33 patients were treated with conventional open surgical repair (Group G), and 52 underwent TEVAR (Group E). The operative indication for TEVAR for CTBAD was a dilated aneurysm > 50 mm in the descending aorta in both groups and an adequate proximal and distal landing zone for endovascular treatment. By contrast, extensive aneurysms involving the entire thoracic and thoracoabdominal aorta and its branches required open repair. The decision to perform TEVAR or open surgical repair was based on the patients' clinical status of multiple preoperative comorbidities including the procedure era. The era of TEVAR was after 2011, whereas open surgical repair was after 2007. Early-, mid-, and long-term results were retrospectively analyzed.

### Surgical procedures

Perioperative cerebrospinal fluid drainage was performed for high-risk cases of paraplegia in both groups before surgery. Group G involved exposure of the thoracic descending and thoracoabdominal aorta through the left thoracotomy (*n* = 25) and thoracoabdominal incision (*n* = 8). All patients in Group G underwent selective ventilation of the single right lung. Partial cardiopulmonary bypass was established via the femoral artery and venous drainage; total cardiopulmonary bypass and circulatory arrest were rarely required. An aortic cross-clamp is usually placed distal to the left subclavian artery. However, if the initial entry was placed near the left subclavian artery, an aortic cross-clamp must be placed between the left common carotid and left subclavian arteries. For proximal and distal anastomosis, a commercial woven Dacron graft was used for central aortic replacement and reinforced with Teflon felt strips. The remaining thoracic aorta was opened; the upper intercostal arteries were ligated. Lower thoracic intercostal arteries were temporally occluded using balloon-tipped catheters. Four cases required intercostal re-implantation with a graft. Some patients underwent surgery under induced hypothermic circulatory arrest using profound hypothermia of 26 ℃. In cases requiring thoracoabdominal replacement, the repair of the visceral and renal artery was reattached, which was usually performed using a Carrel patch directly to the aortic graft. In addition, in all patients in Group G, motor-evoked potentials (MEPs) were used with or without re-implantation of the intercostal arteries.

Regarding Group E, who underwent TEVAR, the therapeutic strategy was to exclude primary entry and those who presented with proximal aneurysms of the descending aorta, in which there were sufficient proximal and distal landing zones, and thus, not off-label use. Depending on the endovascular device used, sheath placement may be required before endograft positioning. Balloon molding of the endograft was avoided. Device oversizing was less than 10%.

### Study variables and definitions

All patient data were retrospectively reviewed. The follow-up was completed with a mean period of 5.8 years (standard deviation [SD] 3.0). Aortic dissection was defined as Stanford classification type B, which is a dissection of the entry site distal to the left subclavian artery. In this study, “chronic” aortic dissection was determined by an interval period over 30 days after symptom onset, based on the International Registry of Aortic Dissection (IRAD) [5].

The diagnosis was based on clinical history and non-invasive imaging study (computed tomography [CT] angiography). Hospital death was defined as death from hospital admission to discharge or within 30 days after surgery. Adverse events as early outcomes were defined as operative death, stroke, paraplegia, or respiratory failure requiring tracheostomy. Stroke was defined as a neurological disorder diagnosed by CT or magnetic resonance imaging after open surgery or TEVAR. Paraplegia was defined as a permanent bilateral motor deficit of the lower extremities.

The primary outcomes were mid- and long-term survival rates, including freedom from all-cause and aorta-related death (ARD). The secondary outcomes were defined as freedom from aortic adverse events in mid- and long-term outcomes. Aortic adverse events included retrograde type A aortic dissection (RTAD), aortoesophageal fistula (AEF), aorto-bronchial fistula (ABF), and thoracic aortic re-intervention. Thoracic aortic re-intervention was defined as additional open or endovascular repair of the descending thoracic and thoracoabdominal aortas due to the progression of aortic disease.

### Statistical analysis

The results for categorical variables are expressed as numbers (percentages of the total). Continuous variables are presented as mean ± SD. The cumulative rate was determined using the Kaplan–Meier method. Logistic regression analysis was used to examine the significance of the clinical, diameter-calculated CT and operative variables. Differences in outcomes were considered statistically significant when *p* < 0.05. The *χ*^2^ test was used to compare categorical variables. The Fisher’s exact test was used if the expected frequency was < 5. Continuous variables were compared using Student’s *t* test. All statistical analyses were performed using JMP version 14.0 (SAS Institute Inc., Cary. NC. USA).

## Results

### Patient characteristics

The baseline characteristics and patient profiles are summarized in Table 1. Comorbidities, such as hypertension, dyslipidemia, diabetes mellitus, chronic kidney disease, coronary artery disease, history of stroke, and prevalence of chronic obstructive pulmonary disease, were not significantly different between the two groups. The patients in Group G were significantly younger than those in Group E, with a significant difference (60.1 ± 14.1 years vs. 68.6 ± 11.6 years, *p* < 0.01). The interval between the onset of TBAD and operation was 4.9 ± 3.9 years in Group G and 1.9 ± 2.9 years in Group E, showing significant differences in each group (*p* < 0.01). A preoperative CT review demonstrated that the maximum diameter of the descending aorta in Group G was larger than that in Group E (G; 59.0 ± 9.3 mm, E; 50.5 ± 7.9 mm, *p* < 0.001), as well as the maximum diameter of the false lumen (G; 35.5 ± 13.8 mm, E; 9.0 ± 10.0 mm, *p* < 0.01).

**Table 1:** Baseline characteristics of the patients

	Group G (*n* = 33)	Group E (*n* = 52)	*P* value
Age	60.1 ± 14.1	68.6 ± 11.6	< 0.01
Male (%)	24 (72.7)	41 (78.9)	0.77
Connective Tissue Disease	3 (7.9)	0 (0.0)	0.12
Hypertension (%)	30 (90.9)	48 (92.3)	0.82
Dyslipidemia (%)	11 (33.3)	22 (44.2)	0.43
Diabetes mellitus (%)	3 (9.1)	11 (21.2)	0.22
Chronic kidney disease (%) (Cr ≧ 1.5 mg/dL)	13 (39.4)	22 (42.3)	0.81
Coronary artery disease (%)	6 (18.2)	8 (15.4)	0.97
Post stroke (%)	3 (9.1)	6 (11.5)	0.96
Chronic obstructive pulmonary disease (%)	5 (15.2)	11 (21.2)	0.57
Interval between onset and operation (year)	4.9 ± 3.9	1.9 ± 2.9	< 0.01
Maximum Diameter (mm)	59.0 ± 9.3	50.5 ± 7.9	< 0.001
Maximum False Lumen Diameter (mm)	35.5 ± 13.8	29.0 ± 10.0	< 0.01

### Perioperative outcomes

All procedures in both groups were technically successful. The thoracoabdominal aortic replacement was performed in eight patients in Group G. Perioperative results are presented in Table 2. Thirty-day mortality was not observed in either group. The overall hospital death rate was only 3.0% (one patient) in Group G due to postoperative low-output syndrome. Two patients (6.0%) in Group G had a stroke, and none in Group E. Paraplegia developed in one patient (3.0%) in Group G and one patient (1.9%) in Group E. Respiratory failure requiring tracheostomy was observed in two patients (6.0%) in Group G, and none in Group E. Group E had better surgical outcomes in terms of fewer complications than did Group G, without finding statistically significant differences. However, in Group E, RTAD occurred in four patients: three were salvageable and one died (Table 3).

**Table 2:** Perioperative Results

	Group G	Group E	*P* value
30-day Mortality (%)	0 (0.0)	0 (0.0)	-
Stroke (%)	2 (6.0)	0 (0.0)	0.30
Paraplegia (%)	1 (3.0)	1 (1.9)	0.66
Respiratory failure (%)	2 (6.0)	0 (0.0)	0.30

**Table 3:** Mid- and long-term results

	Group G	Group E	*P* value
All-cause mortality (%)	8 (24.2)	10 (19.2)	0.58
Aorta-related death (%)	7 (21.2)	7 (13.5)	0.53
Retrograde Type A Aortic Dissection (%)	0 (0.0)	4 (7.7)	0.27
Aorto-Esophageal Fistula (%)	0 (0.0)	2 (3.9)	0.69
Aorto-Brochial Fistula (%)	1 (3.0)	1 (1.9)	0.69
Thoracic Re-intervention (%)	5 (15.2)	16 (30.8)	0.10
Thoracic endovascular repair (%)	5 (15.2)	8(15.4)	0.78
Open Conversion (%)	0 (0.0)	8 (15.4)	0.047

### Mid- and long-term outcomes

**Fig. 1:**
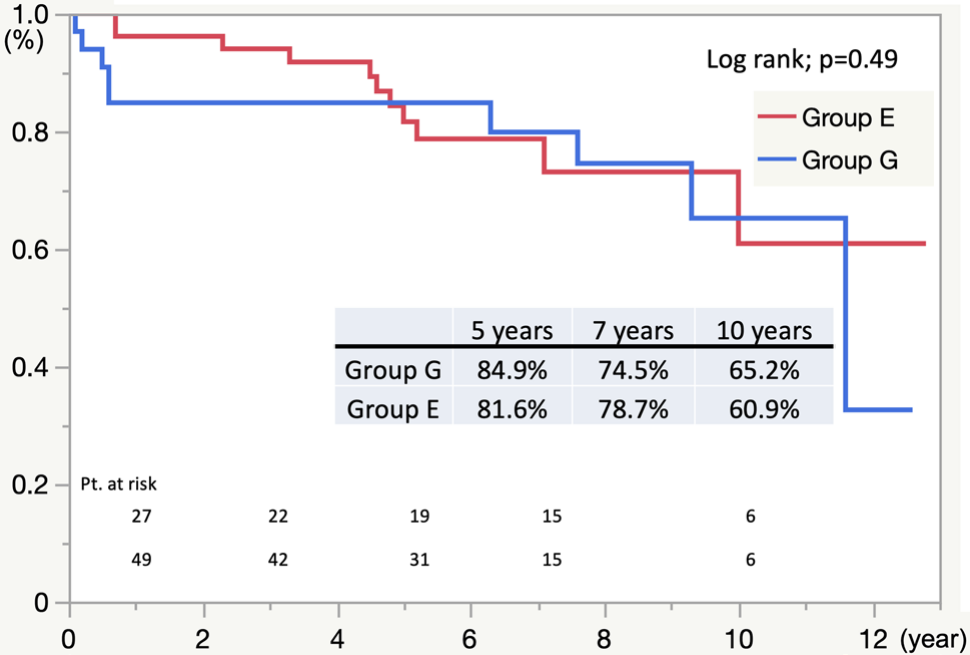
Freedom from All-cause Death

The overall estimated postoperative survivals at 5, 7, and 10 years, as assessed by the Kaplan–Meier method in Group G, were 84.9%, 74.5%, and 65.2%, respectively, and 81.6%, 78.7%, and 60.9%, respectively, in Group E (*p* = 0.49) (Fig. 1). Delayed mortality occurred in two patients who underwent open surgery for stroke, one patient in Group G who had hemoptysis and developed ABF, and seven patients in Group E due to aneurysm rupture (*n* = 3), AEF (*n* = 1), RTAD (*n* = 1), and heart failure (*n* = 1). The cause of death could not be confirmed in one patient.

**Fig. 2:**
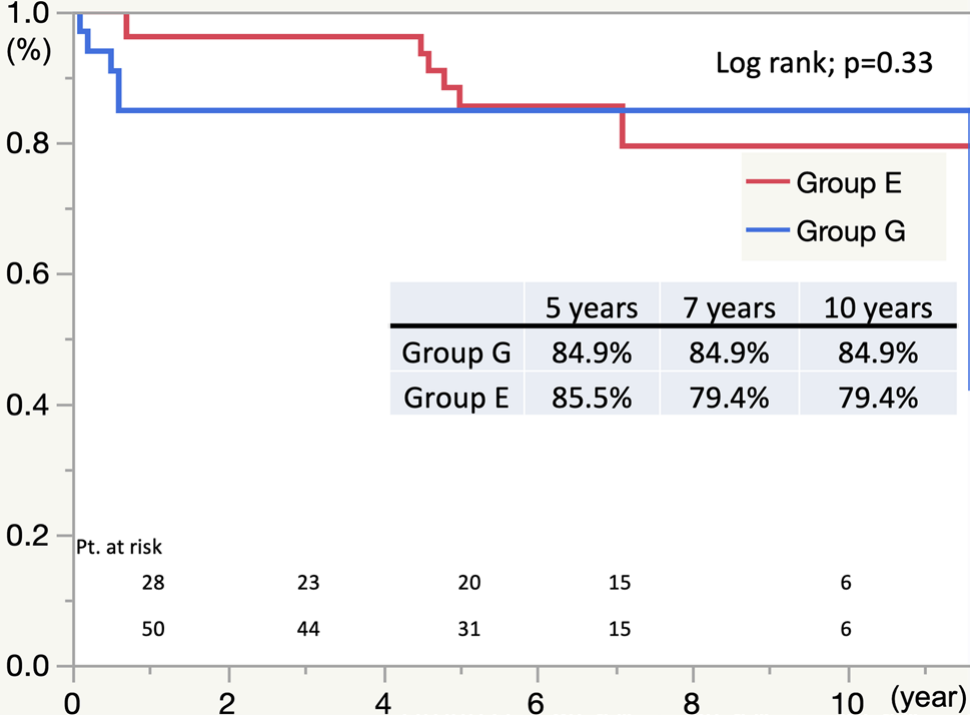
Freedom from Aorta-related Death

The avoidance of aortic-related death was not significantly different between the two groups at 10 years (84.9% in Group G and 74.9% in Group E). However, the rate crossed over at seven years postoperatively (Fig. 2) (*p* = 0.33).

**Fig. 3:**
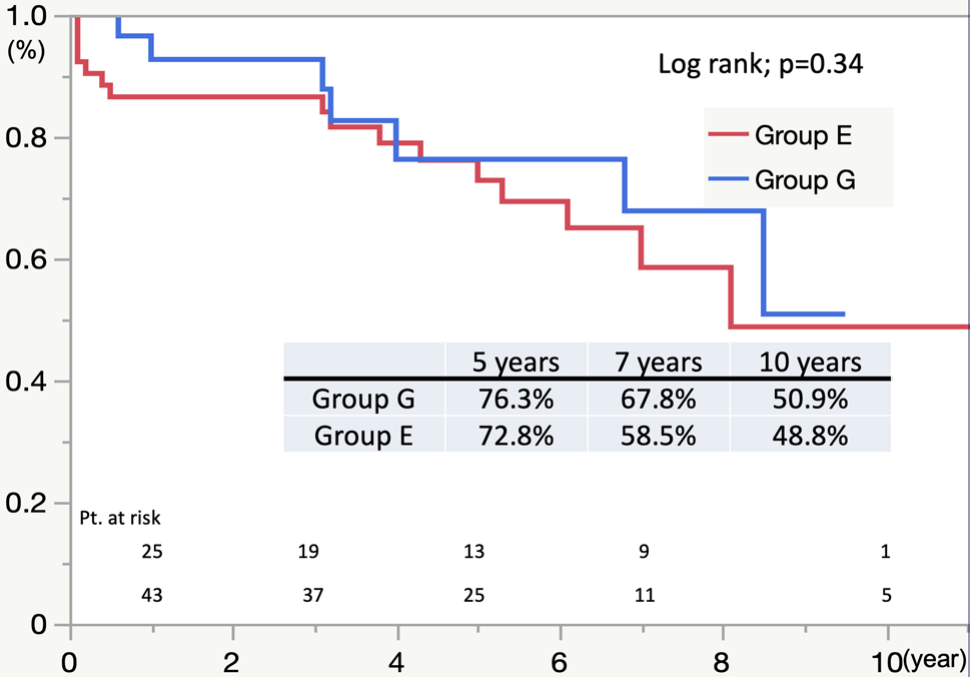
Freedom from Re-intervention

Figure 3 shows the estimated Kaplan–Meier curves of freedom from thoracic re-intervention for Group G and Group E. The overall postoperative freedom from thoracic re-intervention at 5, 7, and 10 years in Group G were 76.3%, 67.8%, and 50.9%, respectively, and 72.8%, 58.5%, and 48.8% in Group E, without a significant difference (*p* = 0.34). The rate of re-intervention was consistently worse in Group E. The indications for re-intervention were pseudo-aneurysm of the proximal site (*n* = 3) and false lumen enlargement in micron graft failure (*n* = 2) in Group G. The causes of late open conversion in Group E were stent-graft infection (*n* = 1), AEF (*n* = 1), type 1a endoleak (*n* = 1), and type 3b endoleak (*n* = 1). The causes of open conversion are shown in Table 4.

**Table 4:** Causes of open conversion

Age	Sex	Indication	Interval (months)	Operation	Outcomes
66	Male	RTAD	1.7	Total Aortic Arch Replacement	Discharge
68	Female	RTAD	0.8	Total Aortic Arch Replacement	Hospital Death
72	Male	RTAD	1.0	Total Aortic Arch Replacement	Discharge
81	Male	RTAD	0.9	Total Aortic Arch Replacement	Discharge
64	Male	Infection	37.4	Descending Aortic Replacement	Discharge
74	Male	AEF	52.1	Descending Aortic Replacement	Hospital Death
59	Male	Type 3b endoleak	60.4	Thoracoabdominal Aortic Replacement	Discharge
68	Male	Type 1a endoleak	74.8	Total Aortic Arch Replacement	Discharge

*AEF* aortoesophageal fistula, *RTAD* retrograde type A aortic dissection

## Discussion

The current gold standard therapy for uncomplicated acute TBAD is optimal medical therapy aiming to limit the progression of dissection by reducing aortic wall pressure. Some reports indicated that patients who did not undergo surgery and received optimal medical treatment had a 90–100% in-hospital survival rates and a 5-year survival of 50–87% [5–11]. In addition, the INSTEAD trial showed complete false lumen thrombosis 2 years after medical treatment in only 19.4% of patients compared with 91.3% in those after TEVAR [7, 12]. However, the effectiveness of conservative therapy in uncomplicated CTBAD remains unclear, which means that it may result in unfavorable long-term outcomes due to severe complications, such as rupture and malperfusion [13]. Desai et al. [14] reported that patients who underwent TEVAR for uncomplicated acute TBAD were successfully treated for the next 10 years but then experienced recurrence. The IRAD recently reported that a mean annual progressive aortic expansion was observed in 59% of patients with medically treated TBAD, with an expansion rate of 1.7 ± 7.6 mm/year [15].

Chronic aortic dissection carries a high risk of late aneurysmal dilation mainly due to false lumen enlargement and rupture. Thus, surgical treatment was indicated. Operative therapy was defined as open or endovascular repair. Endovascular repair has supplanted open surgery for the treatment of the descending aorta, with good short-term results. However, the preference for endovascular surgery for CTBAD remains controversial. Several studies reported in a systematic review that the long-term advantage of endovascular repair over open repair remains unclear and requires further investigation [4, 16, 17].

A careful treatment strategy for CTBAD is required for endovascular repair, which not only targets the occlusion of the proximal entry tear but also of the false lumen to prevent aortic dilation and promote aortic remodeling. Stent graft coverage of the aortic entry tear provides mid- and long-term results of sealing the primary entry, including false lumen thrombosis, which can potentially reduce late ARDs. In addition, devices and delivery systems, as well as an understanding of potential complications, may improve endovascular repair [18].

However, endovascular repair does not deliver the expected results. Guangqi et al. [19] reported that 22 of 121 consecutive patients who underwent endovascular repair of acute and CTBAD had postoperative endoleaks, with a 30-day mortality rate of 8.2%. TEVAR is not risk-free and has a high 30-day mortality rate; severe complications, such as RTAD (2.5–8%) [20, 21], stroke (4.6%), and paraplegia (1.9–4.4%) may also occur [22, 23]. TEVAR in patients with extensive aneurysmal sac may have limitations in terms of aneurysm size and location, occlusion by the false lumen, or thrombus formation for CTBAD [4, 24]. In our study, as presented in Table 4, the causes of open conversion after TEVAR were RTAD during the early postoperative period, infection during the mid-term outcomes, and type 1a and 3b endoleaks during the long-term outcomes. The choice of TEVAR for CTBAD remains controversial because stent grafting bears the risk of eliminating antegrade false lumen flow by persisting through the primary entry. In contrast, retrograde false lumen flow persists through potential dissection re-entry more distally. Regarding CTBAD, false lumen patency can cause worse outcomes. Therefore, TEVAR alone cannot be used in false lumens, which should be treated to promote thrombosis or excluded from systemic circulation [1]. In other words, the treatment of chronic aortic dissection requires intervention in the false lumen, and retrograde false lumen perfusion should be controlled to maintain perfusion and pressurization [24].

Open surgical repair is an acceptable option for most patients with progressive aneurysmal dilation of CTBAD. In our study, there were no ARDs in Group G in the early outcomes, and only seven (21.2%) cases occurred in the mid- and long-term outcomes, which was not significantly different from that in Group E. In addition, few perioperative complications, such as stroke, paraplegia, and respiratory failure, occurred, which was not significantly different from that in Group E. Furthermore, only one patient had fatal complications, such as aorto-bronchial fistula, in the mid- and long-term outcomes. Some reports have mentioned that conventional open surgery is acceptable, with reported in-hospital mortality rates between 9.6 and 22.4% [25, 26] and a 5-year survival rate of 78% in other studies of thoracoabdominal aortic replacements [25, 27]. In patients with chronic aortic dissection, control of blood flow in the false lumen is debatable. Yamana et al. [28] reviewed the mid-term results of the open repair of chronic aortic dissection with anastomosis to the true lumen only and both true and false lumens. They indicated that there were no differences in survival, ARD, late distal aortic events, and false lumen conditions, although thrombosis of the false lumen was better with true lumen only. Nozdrzykowski et al. [24] indicated that the treatment of CTBAD in patients with extensive aneurysms, malperfusion, or acute rupture may be surgically challenging, and the use of TEVAR might be limited in terms of aneurysm size and location, occlusion by the false lumen of the dissection, or thrombus formation within the chronic aneurysm. Fleerakkers et al. [29] reported that in patients with chronic dissections and aneurysmal degeneration, open surgery remains the gold standard, particularly in younger patients with reasonable life expectancy and limited comorbidities. TEVAR or fenestrated and branched endovascular repair can be considered in selecting patients (frail older patients) with suitable anatomy. Our results indicated that open repair CTBAD could be performed safely, although invasive.

However, even using open repair, the risk of re-intervention remains uncertain. Boufi et al. [30] discussed that open repair was not exempt from re-intervention risk. Indeed, in the open group, the rate was as high as 12%, with more than half with the distal expansion of the dissected aorta. However, distal anastomosis is usually performed in the apparently healthy aorta. Ryomoto et al. [31] reported that open surgery is preferable for CTBAD but is associated with late adverse events in the distal unresected aortic portion. In our study, five patients (15.2%) required additional TEVAR for pseudo-aneurysm of the anastomotic site in the mid- and long-term outcomes. The results were similar to those of Group E, in which eight patients (15.4%) required re-intervention. Thus, additional procedures in the mid- and long-term outcomes were similar in both groups.

Intervention for CTBAD could be performed with good results, either using open or endovascular repair. Based on these results, as several other studies reported [29, 32], open repair remains the golden standard for CTBAD, particularly in patients with good life expectancy and limited comorbidities. TEVAR is indicated for high-risk patients for open surgery with anatomic features favorable for endovascular approach.

### Limitations

Our study has several limitations. First, this was a retrospective study. Patient selection bias was not considered, which may have led to partially improved results compared with the anatomical and clinical status. Second, the cohort of treated patients was small. Third, the rates of major adverse outcomes were probably underestimated in both procedures. Furthermore, propensity matching was not performed for patient selection. Particularly, the age of the patients in Group E was significantly higher compared with those in Group G. Several studies have indicated that ages less than 60 years were associated with significantly increased aortic growth rates [1, 33, 34]. Preoperative CT showed that those in Group G had larger aortic aneurysm diameters than those in Group E. Therefore, a comparison with other cohort studies is necessary. Despite these limitations, we suggest that this study could provide a sole comparative assessment in which results were similar between the mid- and long-term outcomes.

## Conclusion

Although endovascular repair of CTBAD is less invasive, the rate of freedom from re-intervention was unsatisfactory. Therefore, the preference for endovascular repair over open repair remains controversial. Some fatal complications, such as RTAD, AEF, and ABF, due to aneurysmal dilation were observed in the endovascular group in the mid- and long-term outcomes. Compared with endovascular repair, open repair can be performed safely without perioperative complications. Open repair has feasible mid- and long-term outcomes.

## Data Availability

The data that support the findings of this study are available from the corresponding author upon reasonable request.
